# Vascular and Macrophage Heme Oxygenase-1 in Hypertension: A Mini-Review

**DOI:** 10.3389/fphys.2021.643435

**Published:** 2021-02-26

**Authors:** Marta Martínez-Casales, Raquel Hernanz, María J. Alonso

**Affiliations:** ^1^Depto. de Ciencias Básicas de la Salud, Facultad de Ciencias de la Salud, Universidad Rey Juan Carlos, Alcorcón, Spain; ^2^Centro de Investigación en Red en Enfermedades Cardiovasculares (CIBER-CV), Madrid, Spain

**Keywords:** hypertension, AngII, macrophages, oxidative stress, inflammation, heme oxygenase-1

## Abstract

Hypertension is one predictive factor for stroke and heart ischemic disease. Nowadays, it is considered an inflammatory disease with elevated cytokine levels, oxidative stress, and infiltration of immune cells in several organs including heart, kidney, and vessels, which contribute to the hypertension-associated cardiovascular damage. Macrophages, the most abundant immune cells in tissues, have a high degree of plasticity that is manifested by polarization in different phenotypes, with the most well-known being M1 (proinflammatory) and M2 (anti-inflammatory). In hypertension, M1 phenotype predominates, producing inflammatory cytokines and oxidative stress, and mediating many mechanisms involved in the pathogenesis of this disease. The increase in the renin–angiotensin system and sympathetic activity contributes to the macrophage mobilization and to its polarization to the pro-inflammatory phenotype. Heme oxygenase-1 (HO-1), a phase II detoxification enzyme responsible for heme catabolism, is induced by oxidative stress, among others. HO-1 has been shown to protect against oxidative and inflammatory insults in hypertension, reducing end organ damage and blood pressure, not only by its expression at the vascular level, but also by shifting macrophages toward the anti-inflammatory phenotype. The regulatory role of heme availability for the synthesis of enzymes involved in hypertension development, such as cyclooxygenase or nitric oxide synthase, seems to be responsible for many of the beneficial HO-1 effects; additionally, the antioxidant, anti-inflammatory, antiapoptotic, and antiproliferative effects of the end products of its reaction, carbon monoxide, biliverdin/bilirubin, and Fe^2+^, would also contribute. In this review, we analyze the role of HO-1 in hypertensive pathology, focusing on its expression in macrophages.

## Introduction

Hypertension is an important risk factor that significantly contributes to worldwide cardiovascular morbidity and mortality. Despite its prevalence and clinical importance, its origin, in many cases, remains unclear, although the role of angiotensin II (AngII) in its pathophysiology is well known. Thus, AngII, via AT_1_ receptor, is associated with cell growth, inflammation, vasoconstriction, apoptosis, and production of extracellular matrix components and reactive oxygen species (ROS) (Kim et al., 2011; Savoia and Volpe, 2011); moreover, AngII also recruits monocytes and other inflammatory cells in heart, vasculature, and kidney during hypertension (Rucker and Crowley, 2017).

Recently, a relationship between inflammation and hypertension-associated damage has been reported. Thus, both the adaptive immunity (Xiao and Harrison, 2020) and cells from the innate immune system, such as macrophages, have been described to be involved in hypertension. Immune cells infiltrate vessels, kidneys, heart, and brain, producing proinflammatory cytokines, and chemokines (Norlander et al., 2018; Caillon et al., 2019). The infiltrating macrophages can amplify local ROS levels, promoting inflammation via activation of redox-sensitive transcription factors, mainly NFκB, leading to inflammasome activation (Xiao and Harrison, 2020). A low degree of inflammation facilitates vascular oxidative stress and decreases nitric oxide (NO) bioavailability, leading to the vascular alterations accounting for the increased peripheral vascular resistance (Norlander et al., 2018; Caillon et al., 2019). Specifically, increased macrophage infiltration has been observed in different hypertension models (Norlander et al., 2018; Caillon et al., 2019) and a causal role of monocytes and macrophages in the hypertension development and the associated vascular alterations has been described (De Ciuceis et al., 2005).

Within the inflammatory processes involved in hypertension, vascular damage due to oxidative stress is of great importance. ROS are mainly produced in the mitochondria and by NADPH oxidase, but also by uncoupled NO synthase and xanthine oxidase. These sources are activated in endothelial, vascular smooth muscle (VSMC), neuronal, and renal tubular cells (Xiao and Harrison, 2020). Oxidative stress promotes endothelial dysfunction and induces proinflammatory monocyte adhesion via increased expression of adhesion molecules (Kumar and Bandyopadhyay, 2005). Oxidative stress also activates cyclooxygenases (COX) generating prostaglandins and thromboxanes, which contribute to vascular alterations and enhances inflammatory responses (Montezano et al., 2015). Additionally, inflammation and oxidative stress can also induce vascular remodeling, with elevated media/lumen ratio, and increase stiffness in hypertension (Hernanz et al., 2014).

Heme oxygenase-1 (HO-1) catalyzes degradation of the pro-oxidant heme generating carbon monoxide (CO), biliverdin (BV), and ferrous iron (Fe^2+^), which are antioxidant and anti-inflammatory. HO-1 has a protective role in hypertension by reducing end organ damage and blood pressure, not only by its expression in several tissues, but also by modulating macrophage polarization toward anti-inflammatory phenotype (Yang et al., 2004; Wenzel et al., 2015; Bellner et al., 2020). This review will describe the role of HO-1 and its enzymatic products in hypertension, focusing on its expression in macrophages.

## Macrophages in Hypertension

Macrophages are the most abundant immune cells in tissues, including vessels, heart, and kidneys. They display remarkable plasticity, which is manifested by a functional and phenotypic differentiation called polarization (Harwani, 2018). Macrophages are usually classified into M1 and M2, with M1 being proinflammatory by producing cytokines such as interleukin-1 beta (IL-1β) or tumor necrosis factor-α (TNF-α), and ROS, and M2 being anti-inflammatory by secreting IL-10 and transforming growth factor-beta (TGF-β). However, classifying macrophages is not so easy, since the great variety of stimuli they receive will give rise to numerous subpopulations (Harwani, 2018).

The M1/M2 macrophage ratio seems to play an important role in the hypertension pathophysiology. Thus, M2 markers are reduced in SHR liver, which contributes to hypertension; furthermore, M2 macrophage induction would normalize blood pressure in established hypertension (Ndisang and Mishra, 2013). In AngII-infused mice, the great vascular M1 infiltration is involved in endothelial dysfunction and hypertension (Gomolak and Didion, 2014). Besides the direct effects of M1 macrophage-produced ROS and inflammatory cytokines, they also affect NO levels. Thus, M1 macrophages increase NO through inducible NO synthase (iNOS) (DeGeorge et al., 1997), which, along with ROS, results in reactive nitrogen species formation, decreasing NO bioavailability and aggravating cellular damage (Hsieh et al., 2014). Therefore, the increased M1/M2 macrophage ratio participates in hypertension, although the cause of this imbalance remains unclear (Harwani, 2018).

In hypertension, AngII, through AT_1_ receptors, drives to differentiation, mobilization, and activation of proinflammatory monocytes into the heart, vessels, and kidney. The M1 macrophages that accumulated in renal interstitium migrate to vascular subendothelium and then produce inflammatory cytokines and ROS, which lead to kidney fibrosis and vascular injury. However, AT_1_ receptor activation suppresses macrophage M1 polarization and reduces the AngII-caused end organ damage (Rucker and Crowley, 2017).

The increased sympathetic activity observed in hypertension also contributes to macrophage polarization and mobilization, as part of neuroimmune interaction (Harwani, 2018). Thus, activation of splenic sympathetic nerve in response to AngII infusion into the central nervous system increases M1 proinflammatory cytokines in some immune reservoirs, such as spleen; in fact, sympathetic innervation of spleen is required for AngII-induced hypertension. Increased renal sympathetic nerve activity also participates in macrophage activation (Harwani, 2018).

## Heme Oxygenases

**FIGURE 1:**
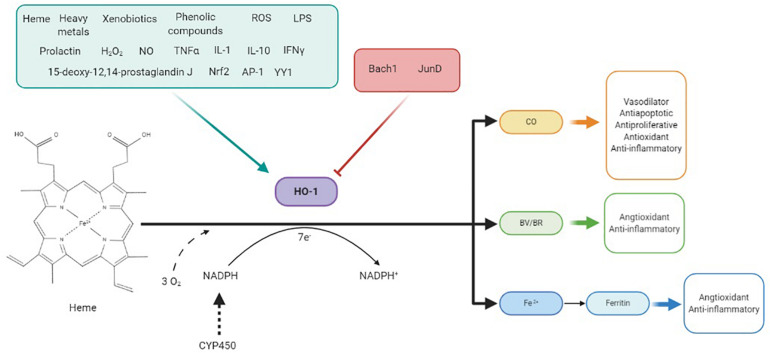
HO-1 is induced by a variety of stimuli such as its substrate heme, heavy metals, xenobiotics, growth factors, or cytokines and repressed by some factors like Bach-1 and Jun D. This enzyme catalyzes the degradation of heme in biliverdin (BV), which is reduced to bilirubin (BR), CO, and Fe^2+^ in equimolar quantities, consuming three molecules of O_2_ per mole of heme and 7 e^–^donates by NADPH through CYP450 system. BV, CO, and Fe^2+^, through ferritin production, have shown to be responsible for antioxidant and anti-inflammatory effects of HO-1. CO also has vasodilator, antiapoptotic, and antiproliferative effects at the vascular level.

Heme oxygenases (HO) are the rate-limiting enzymes in heme catabolism, regulating its intracellular levels (Figure 1). These enzymes catalyze degradation of heme *b* to equimolar quantities of the bile pigment BV, CO, and Fe^2+^ (Kim et al., 2011; Ayer et al., 2016). Thereafter, BV reductase (BVR) reduces BV to bilirubin (BR), which combines with UDP-glucuronytransferase and is excreted in the bile (Abraham and Kappas, 2008). Additionally, HO recycle iron from senescent erythrocytes and extrahematopoietic cells, explaining their high basal activity in tissues rich in reticuloendothelial cells (Abraham and Kappas, 2008).

HO are evolutionarily highly conserved enzymes (Ayer et al., 2016) located in microsomes (Maines et al., 1977) and mitochondria (Di Noia et al., 2006) of all tissues. In mammals, HO family consists of two enzymes, HO-1 and HO-2, with a molecular weight of 32 and 36 kDa, respectively, a third 33 kDa enzyme, HO-3, was also detected, but finally, it has been proved to be a pseudogene derived from the HO-2 transcript (Abraham and Kappas, 2008; Loboda et al., 2008). Both HO-1 and HO-2 contain a sequence of 24 amino acids, the “heme binding pocket,” which allows them to bind to the heme group, and a hydrophobic region at the -COOH terminus that acts as anchorage to the endoplasmic reticulum membrane (Ayer et al., 2016). HO-1 is inducible; therefore, it is generally undetected under normal conditions, except in tissues with a high rate of degradation of senescent red blood cells, where it predominates even under unstressed conditions (Loboda et al., 2008; Ayer et al., 2016). Furthermore, high HO-1 levels are present in macrophages, mainly responsible for heme degradation in these cells (Kartikasari et al., 2009). Conversely, HO-2 is constitutive, being highly present in testes and brain (Durante et al., 1997). Although both isoforms are involved in antioxidant defense, inflammatory response regulation, and cell proliferation, they differ in their physiological and biochemical properties; thus, HO-1 is involved in iron homeostasis, angiogenesis, mitochondrial function, and innate and adaptive immunity regulation, while HO-2 is involved in oxygen and redox sensing, neovascularization, and neuroprotection (Ayer et al., 2016). That is, HO-2 is the physiological regulator of cellular functions, while HO-1 has a cytoprotective role, regulating tissue responses to injury in pathophysiological states (Kim et al., 2011); therefore, this review will be mainly focused on vascular and macrophage HO-1 and its possible role in hypertension-associated vascular alterations.

HO-1 expression is regulated by many endogenous and exogenous stimuli, including its natural substrate heme, heat, heavy metals, xenobiotics, TNF-α, growth factors, IL-1, IL-10, interferon gamma, lipopolysaccharides, NO, hydrogen peroxide (H_2_O_2_), or phenolic compounds such as curcumin (Figure 1). These stimuli induce the expression of HO-1 by transcription factors such as Nrf2, AP-1, or YY1, although factors such as Bach-1 or JunD repress its expression (Figure 1; Loboda et al., 2008; Ayer et al., 2016). One of the most important roles of HO-1 is heme availability regulation. Heme is a prosthetic group for fundamental proteins such as hemoglobin, myoglobin, cytochromes, HO-1, catalases, or peroxidases, in addition to important enzymatic systems in hypertension, such as COX or NOS (Kumar and Bandyopadhyay, 2005; Loboda et al., 2008); however, heme can also be harmful once released from hemoproteins. At the vascular level, free heme is toxic, increasing the oxidant state by amplifying radical species production (Balla et al., 1993). Additionally, its presence enables the synthesis of enzymes such as COX-2, cytochrome P450, and iNOS, and then contributing to inflammation and ROS production; therefore, HO-1 allows reduction of endothelium-derived contracting factors, such as endoperoxides, thromboxanes, and the cytochrome P450-derived eicosanoid 20-HETE, as well as of the excessive iNOS-derived NO (Abraham and Kappas, 2008).

## Vascular and Macrophage HO-1 and Its Role in Hypertensive Alterations

HO-1 is induced by oxidant stress, and its upregulation of HO-1 response is associated with cyto- and tissue protection against pro-oxidant and proinflammatory conditions in may human diseases (Kim et al., 2011). Thus, its deficiency has detrimental effects as enhanced systemic inflammation, abnormalities of coagulation/fibrinolysis system, or vascular endothelial injury (Chen et al., 2013; Loboda et al., 2016). Upregulation of HO-1 prevents vascular dysfunction and endothelial cell death through decrease in ROS levels (Abraham and Kappas, 2008). It is also involved in vasodilation, participates in angiogenesis and vasculogenesis (Bussolati and Mason, 2006), and has immunomodulatory effects, which may be beneficious against the inflammation observed in different cardiovascular diseases (Chen et al., 2013; Vijayan et al., 2018). The presence of a microsatellite polymorphism of (GT)_*n*_ repeats in human HO-1 promoter is relevant in the development of various clinical conditions, particularly cardiovascular diseases. Thus, long (GT)_*n*_ sequences (>25) are associated with weak HO-1 transcription, while short (GT)_*n*_ fragments are linked to low plasma levels of inflammation markers. Hence, patients with long (GT)_*n*_ fragments have increased susceptibility to cardiovascular diseases (Abraham and Kappas, 2008; Loboda et al., 2008; Ayer et al., 2016; Vijayan et al., 2018), including hypertension (Wenzel et al., 2015).

Regarding hypertension, some authors observed increased HO-1 levels in aorta of DOCA-salt (Nath et al., 2007) and SHR rats (Cheng et al., 2004). Additionally, a relationship between AngII and HO-1 levels has been reported, with increased AngII-induced HO-1 expression in kidney (Aizawa et al., 2000), heart (Ishizaka et al., 2000), and aorta (Ishizaka et al., 1997). HO-1 is expressed in adventitial and VSMC from normotensive animals, but not in endothelial cells; however, AngII-infused mice presented increased vascular HO-1 mRNA, protein, and activity mostly in endothelium and adventitia (Ishizaka et al., 1997), while VSMC stimulated with AngII showed HO-1 levels of downregulation (Ishizaka and Griendling, 1997). HO-1 induction in adventitial and endothelial cells might try to counteract AngII-induced oxidative stress and inflammation, playing an important role in blood pressure regulation and vascular homeostasis, although the increased HO activity is insufficient to compensate the damage (Tiwari and Ndisang, 2014). Therefore, HO-1 is an important blood pressure regulator in different hypertension models. Thus, in SHR, the chemical induction of HO-1, the administration of its substrates, and HO-1 gene transfer attenuate hypertension, an effect that is repressed by inhibitors of this enzyme (Levere et al., 1990; Sabaawy et al., 2001; Wang et al., 2006; Li et al., 2013) and that has been associated to the improvement of endothelial dysfunction by mechanisms involving EDH-type relaxations (Li et al., 2013). Similarly, in AngII-induced hypertension, the HO-1 inducer cobalt protoporphyrin-IX and the widespread transgenic expression of human HO-1 reduce blood pressure (Yang et al., 2004; Vera et al., 2007). Furthermore, endothelial-specific expression of HO-1 attenuates AngII-induced hypertension and the associated vascular dysfunction, by increasing p-eNOS and reducing oxidative stress and inflammatory cytokine levels (Cao et al., 2011).

By using AngII-infused HO-1-deficient mice, Wenzel et al. (2015) proposed that HO-1 regulates vascular function, not only by its vascular expression, but also by shifting circulating and infiltrating macrophage toward the anti-inflammatory phenotype, with possible implications for all-cause mortality; additionally, monocytic HO-1 mRNA levels are positively associated with endothelial function in hypertensive patients (Wenzel et al., 2015). As mentioned, HO-1 shifts macrophages to the anti-inflammatory phenotype (Wenzel et al., 2015; Vijayan et al., 2018; Bellner et al., 2020), although this phenotype would not be the classic M2, but a different type known as M-hem; this is characterized by increased intracellular iron levels and upregulated HO-1 and IL-10 expression along with decreased inflammatory activation (Boyle, 2012; Boyle et al., 2012). Therefore, HO-1 expression in macrophages seems to have a beneficial effect by reducing inflammation in hypertension target organs (Wenzel et al., 2015; Bellner et al., 2020). However, although HO-1 expression is increased in the adventitia of hypertensive rats, the presence of macrophages in this vascular layer cannot explain the staining observed for HO-1 (Ishizaka et al., 1997).

When referring to the beneficial effects of HO-1, mention should be made to its enzymatic end products CO, Fe^2+^, and BV, since they have shown to be responsible for many of these effects, as described below (Figure 1).

### Carbon Monoxide

CO is the more relevant HO-1 end product because of its role in hemodynamic regulation having several actions. Thus, CO prevented the AngII-induced increased ROS formation, CCR2 expression, and chemotactic activity of human monocytes and inhibited the blood pressure increase (Johnson et al., 1995; Morita et al., 2003).

CO induces vasodilation by activating soluble guanylate cyclase (Durante et al., 1997) and calcium-activated K^+^ channels in smooth muscle cells (Wang and Wu, 1997); therefore, HO-1-derived CO release contributes to endothelium-dependent vasodilation (Durante et al., 1997). Moreover, CO inhibits constrictor responsiveness to myogenic stimuli and attenuates the renal arteries’ sensitivity to vasoconstrictors, thus contributing to regulate the pressor responsiveness to AngII (Kozma et al., 1999; Kaide et al., 2001).

Furthermore, CO shows anti-apoptotic effects in endothelial and VSMC, through p38-MAPK and cGMP, respectively, and antiproliferative effect in VSMC by inhibiting ERK (Brouard et al., 2002; Liu, 2002; Song et al., 2002). Another important role of CO is its anti-inflammatory action. In macrophages, CO downregulates proinflammatory cytokine production, including TNF-α, IL-1β, and macrophage inflammatory protein-1β (MIP-1β); simultaneously, CO increases IL-10 expression, leading to anti-inflammatory tissue protection, which is dependent on the modulation of mitogen-activated protein kinase (MAPK) activities (Otterbein et al., 2000). CO also regulates proinflammatory transcription factors, such as NF-κB and AP-1 (Sarady et al., 2002; Morse et al., 2003). Likewise, in macrophages, CO downregulates the ROS-dependent recruitment of TLR4 to the plasma membrane (Otterbein et al., 2000).

### Biliverdin and Bilirubin

BV and BR are antioxidants, which may downregulate the redox mechanisms involved in AngII vascular actions (Yang et al., 2004); in fact, BR is one of the most powerful plasma scavenger of ROS and RNS (Jansen et al., 2010). BR may reduce the hypertension severity and elicits cytoprotection by lowering oxidative stress, preventing vascular NADPH oxidase activation, inhibiting lipid peroxidation and peroxynitrite-mediated oxidations, protecting against H_2_O_2_ toxicity, increasing NO half-life, and inhibiting iNOS (Kwak et al., 1991; Minetti et al., 1998; Wang et al., 2004). Moreover, BR also blocks key events in inflammation and then abrogates the inflammatory response (Sarady-Andrews et al., 2005). In this sense, the interference with leukocyte adhesion to vascular endothelium, via changes in adhesion molecule expression observed by HO-1 upregulation, has been attributed to BV and/or BR (Hayashi et al., 1999; Vachharajani et al., 2000).

The antioxidant and anti-inflammatory actions of BR might explain the inverse relationship between plasma BR levels and systolic blood pressure (Chin et al., 2009; Wang and Bautista, 2015). However, the BR effect on systolic blood pressure and hypertension was relatively weak (Wang and Bautista, 2015), and some studies conducted in SHR have even shown no reduction in blood pressure due to BR, attributing this effect to CO (Ndisang et al., 2002).

BV has less antioxidant activity than BR, but induces BVR phosphorylation, allowing in macrophages PI3K-Akt-IL-10 activation, thus exerting anti-inflammatory action (Wegiel et al., 2009). Moreover, this enzyme inhibits TLR4 by binding directly to the TLR4 promoter, increasing its anti-inflammatory activity (Wegiel et al., 2011).

### Fe^2+^

Another resulting product from heme degradation by HO-1 is Fe^2+^, which generates ROS through Fenton reaction and is toxic for endothelial cells by enhancing oxidant damage (Balla et al., 1993; Berberat et al., 2003). However, the increased iron and CO produced by HO-1 activity is associated with increased levels of ferritin through its regulatory protein binding and by activation of iron response elements (Balla et al., 1992; Wu and Wang, 2005). Ferritin is a protective enzyme that sequesters Fe^2+^, protects endothelial cells from iron-induced oxidative stress and from ultraviolet light, and is also an endothelial cytoprotective antioxidant, presumably due to the inhibition of TNF-α-induced apoptosis (Berberat et al., 2003; Abraham and Kappas, 2008). Moreover, ferritin also exerts anti-inflammatory effects (Bolisetty et al., 2015) and, in addition to sequester iron, it can bind free heme, reducing its bioavailability (Kadir et al., 1992). We can speculate that these protective effects of ferritin in endothelium could have a beneficial role reducing hypertensive-associated alterations caused by oxidative stress and inflammation.

## Conclusion

Oxidative stress and inflammation highly contribute to hypertensive alterations, and macrophage polarization to inflammatory phenotype plays a key role in those processes. HO-1, the inducible isoform of the heme-degrading enzyme HO, is activated in response to oxidative and inflammatory stimuli in an attempt to counteract tissue insults. The HO-1 effect is mediated by regulating levels of heme, which has potential pro-oxidant and proinflammatory effects, as well as through the action of its end products CO, BV/BR, and Fe^2+^. At the vascular level, HO-1 and its end products exert antioxidant, anti-inflammatory, vasodilator, antiapoptotic, and antiproliferative effects. In macrophages, HO-1 expression shifts their phenotype to anti-inflammatory, which is related to improvement of vascular function and blood pressure. In spite of the beneficial effects derived from HO-1 induction in hypertension, this is not sufficient to compensate for the damage of hypertensive pathology. Thus, the use of pharmacological agents that potentiate this system could constitute a good therapy for the treatment of hypertension.

## Author Contributions

MJA and RH conceived the manuscript and revised it critically. MM-C drafted the manuscript and prepared the figure. All authors contributed to the article and approved the submitted version.

## Conflict of Interest

The authors declare that the research was conducted in the absence of any commercial or financial relationships that could be construed as a potential conflict of interest.
